# Frequency down-conversion of terahertz waves at optically induced temporal boundaries in GaAs waveguides

**DOI:** 10.1515/nanoph-2024-0010

**Published:** 2024-05-20

**Authors:** Keisuke Takano, Satoko Uchiyama, Shintaro Nagase, Yuka Tsuchimoto, Toshihiro Nakanishi, Yosuke Nakata, Joel Pérez-Urquizo, Julien Madéo, Keshav M. Dani, Fumiaki Miyamaru

**Affiliations:** Department of Physics, Faculty of Science, 13056 Shinshu University, Nagano 390-8621, Japan; Department of Electronic Science and Engineering, 74062 Kyoto University, Kyoto 615-8510, Japan; Graduate School of Engineering Science, 320550 Osaka University, Osaka 560-8531, Japan; Center for Quantum Information and Quantum Biology, 320550 Osaka University, Osaka 560-0043, Japan; Femtosecond Spectroscopy Unit, 508336 Okinawa Institute of Science and Technology Graduate University, Okinawa 904-0495, Japan; Laboratoire de Physique de l’École Normale Supérieire ENS, 27063 Université PSL, CNRS, Sorbonne Université , Université de Paris, F-75005 Paris, France

**Keywords:** time-varying medium, temporal boundary, frequency conversion, terahertz wave, frequency division multiplexing

## Abstract

In this study, the frequency down-conversion of terahertz waves is analytically and experimentally demonstrated at the temporal boundaries within a GaAs waveguide. The temporal boundary is established by photoexciting the top surface of the waveguide, thereby instantaneously increasing its electrical conductivity. This photoexcited waveguide supports a transverse electromagnetic (TEM) mode with a frequency lower than those of the transverse magnetic (TM) modes present in the original waveguide. At the temporal boundary, the incident TM mode couples with the TEM mode, resulting in frequency down-conversion. Subtracting the propagation loss from the frequency-converted components indicates that the frequency conversion occurs with an efficiency consistent with the analytical predictions. The propagation loss is primarily due to ohmic loss, caused by the finite electrical conductivity of the photoexcited region. Given that the frequency of transverse electric modes is up-converted at the temporal boundary, our findings suggest that the direction of frequency conversion (upward or downward) can be controlled by manipulating the incident polarization. The polarization-dependent frequency conversion in waveguides holds significant potential for applications in devices designed for the interconversion of terahertz signals across various frequency channels. This capability is instrumental in the development of frequency-division-multiplexed terahertz wave communication systems, thereby enabling high data transfer rates.

## 1 Introduction

With the ever-increasing demand for information transfer, expectations for broadband and high-speed data transmission technologies utilizing terahertz waves are continuously rising [1]. Frequency division multiplexing techniques play a pivotal role in optimizing the use of frequency resources in the terahertz band, thereby enhancing the rate of information transfer [2], [3], [4]. The interconversion between arbitrary-frequency channels necessitates both up- and down-conversion of frequencies [5].

Typically, two conventional approaches are utilized for frequency conversion. The first is the electronics approach, which employs diodes with nonlinear responses [6], [7], [8], and the second is the photonics approach, utilizing the nonlinear optical effects of crystals [9], [10], [11]. In both approaches, the output frequency is constrained to multiples of the input frequency when considering single-frequency excitation. In particular, frequency down-conversion to a non-zero frequency necessitates a complex system that includes an additional input of the exact frequency difference between the desired and fundamental frequencies. Additionally, achieving high conversion efficiency in frequency conversion devices for terahertz waves is challenging. In the electronics approach, the response of the circuits limits the generation of high-frequency terahertz waves. The conversion efficiency obtained from nonlinear optical effects, such as harmonic generation and difference-frequency generation in photonics, is dependent on the intensity of the input waves, rendering them unsuitable for the terahertz frequency range due to the challenge of obtaining intense terahertz sources.

Frequency conversion in time-varying systems [12] presents an alternative approach for realizing highly efficient, arbitrary frequency up- and down-conversion of terahertz waves without the need for difference-frequency inputs. Compared with time-invariant systems, the temporal variation of material parameters, such as the refractive index, enables frequency conversion. Initial studies on frequency changes in time-varying systems demonstrated the frequency up-conversion of microwaves in rapidly growing plasmas [13], [14], [15]. Subsequent frequency conversion in the optical frequency range was achieved using semiconductor waveguides with mechanically movable components [16], [17] and carrier generation through optical excitation [18], [19]. In the terahertz frequency range, which lies between the microwave and optical frequencies, the temporal manipulation of plasma frequency in semiconductors has been applied to frequency up-conversion [20]. Frequency conversion using easily manageable planar metamaterial devices has increased the flexibility of the conversion frequency [21], [22].

Performing frequency conversion for terahertz waves in waveguides is highly desirable due to its compatibility with future terahertz wave circuits [23]. The mechanical deformation of waveguides has been utilized for the frequency up- and down-conversion of infrared light [17]. However, it is difficult to induce larger frequency shifts relative to frequencies in THz by mechanical deformation of waveguides to fully take advantage of broad terahertz frequency range. Miyamaru et al. demonstrated the frequency up-conversion of terahertz waves in a GaAs waveguide [24]. In their experiment, a terahertz wave packet with a transverse electric (TE) mode was excited in a GaAs waveguide featuring a metal-coated bottom surface. As the terahertz wave packet propagated through the waveguide, optical pumping at the waveguide’s top surface generated conduction electrons, altering the structure to a double-metalized (parallel plate) configuration. This photoexcitation-induced transformation occurred instantaneously for the terahertz waves, with the altered structure persisting beyond the initial change. This transition was conceptualized as a temporal boundary, analogous to a spatial boundary, facilitating the frequency up-conversion in the TE mode. However, frequency down-conversion presents greater challenges than up-conversion and has not yet been demonstrated. Typically, frequency down-conversion is improbable at the photoinduced temporal boundary in waveguides, as the boundary condition tends to induce more spatially confined propagation modes with higher frequencies post-photoexcitation. The waveguide structures and mode conditions that allow frequency down-conversion despite the confinement of electromagnetic fields by photoexcitation has been counterintuitive and non-trivial. To overcome this challenge, we have exploited the polarization degree of freedom.

In this study, we have both theoretically and experimentally demonstrated the frequency down-conversion of transverse magnetic (TM) mode terahertz waves in a GaAs waveguide. Unlike the TE modes, the incident TM modes can couple with the transverse electromagnetic (TEM) mode of the double-metalized waveguide at the temporal boundary. Since the dispersion curve of the TEM mode is situated at a lower frequency than that of the incident TM mode, frequency down-conversion can occur at the temporal boundary. We have estimated the efficiency of the frequency conversion for the TM modes theoretically and observed the frequency down-conversion of the terahertz waves experimentally. By subtracting the propagation loss from the frequency-converted terahertz waves, we determined that the efficiency of the frequency conversion process aligns with the analytical predictions, thereby validating the proposed theory. Variations in the intensity of the pump pulse revealed that the intensity of the frequency-converted terahertz waves is primarily influenced by Ohmic loss due to the finite electrical conductivity of the optical pump region. Considering the frequency up-conversions for TE mode terahertz waves [24], the direction of frequency conversion (either up or down) can be selectively controlled by manipulating the incident polarization.

## 2 Theory

**Figure 1:**
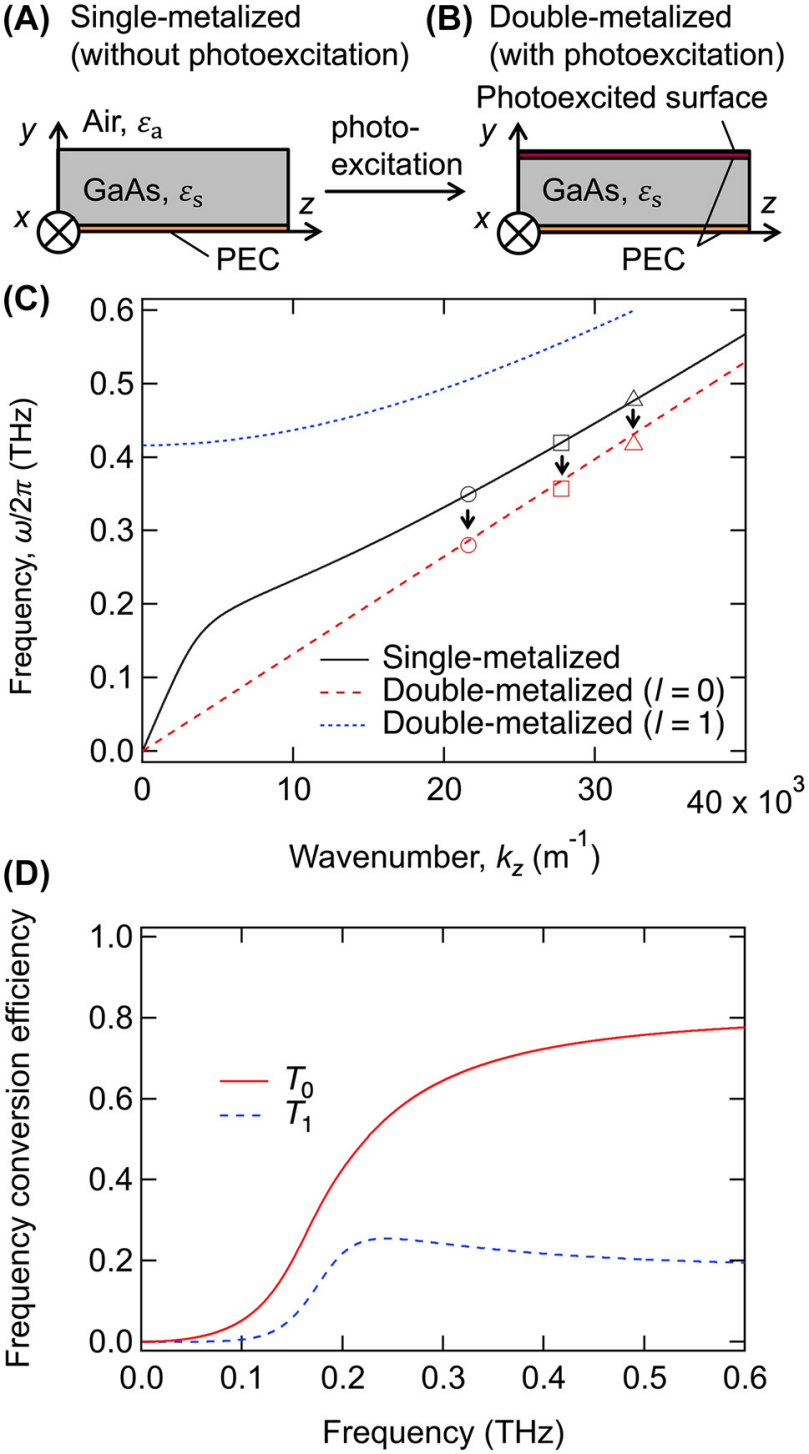
Schematics of the (A) single-metalized (without photoexcitation) and (B) double-metalized (with photoexcitation) waveguides. (C) Dispersion curves of transverse magnetic (TM) and transverse electromagnetic (TEM) modes in the single- and double-metalized waveguides. In this analysis, the thickness of the waveguide is *d* = 100 μm, and the relative permittivity of GaAs is *ɛ*_s_ = 12.96. The input and output frequencies observed in the experiment are plotted using red and black markers, respectively; the circles, squares, and triangles correspond to the frequency conversion for input frequencies of 0.35, 0.42, and 0.48 THz, respectively. (D) Energy efficiency of the frequency conversion *T*_*l*_ from the lowest TM mode in the single-metalized waveguide to the TEM (*l* = 0) and TM (*l* = 1) modes in the double-metalized waveguide.

In the context of a homogeneous medium experiencing a frequency shift at a temporal boundary, where there’s a change in the refractive index, the transmission and reflection coefficients have previously been derived based on the continuity of electromagnetic fields at the boundary [25], [26], [27]. By contrast, in this study, we focus on terahertz waves propagating in a waveguide, with a temporal boundary induced by photoexcitation leading to changes in the waveguide structure and the associated dispersion relation. Figure 1(A) illustrates the schematic of the waveguide structure designed for the frequency down-conversion of terahertz waves. The bottom surface of the GaAs slab waveguide is metal-coated. Concurrently, as the terahertz waves propagate along the *z*-direction of the waveguide, its top surface undergoes photoexcitation. For electromagnetic waves whose frequencies are significantly lower than the plasma frequency of the excited area, this configuration effectively becomes a parallel metallic plate waveguide, as depicted in Figure 1(B). The act of photoexcitation creates a temporal boundary, transitioning the waveguide structure from the setup shown in Figure 1(A) to that in (B). In this section, we delve into the wave scattering phenomenon at the temporal boundary in the waveguide, by connecting the electromagnetic modes present at the boundary.

### 2.1 Dispersion relations involved in the frequency conversion

First, we derived the dispersion relations of the propagation modes in the semiconductor dielectric waveguides to assess the feasibility of utilizing the TEM mode for frequency down-conversion. Prior to the temporal boundary, the terahertz waves propagate into the single metalized waveguide (Figure 1(A)). The lowest-order mode of the TM electromagnetic wave (*H*_*z*_ = 0) was considered to travel in the *z*-direction in the GaAs waveguide. The thickness of the waveguide was *d* = 100 μm, and the bottom surface (*y* = 0) was covered with metal. For simplicity, the metal was assumed to be a perfect electric conductor (PEC), and the waveguide was uniform in the *x*-direction. The permittivity inside and outside the waveguide was assumed to be *ɛ* = *ɛ*_s_ *ɛ*_0_ for 0 ≤ *y* ≤ *d* and *ɛ* = *ɛ*_a_ *ɛ*_0_ for *y* > *d*, with the relative permittivity of GaAs *ɛ*_s_ = 12.96, the relative permittivity of air *ɛ*_a_ = 1, and the permittivity of a vacuum *ɛ*_0_. The permeability was assumed to be equal to the permeability of a vacuum *μ*_0_. The electromagnetic waves in the waveguide propagated with a factor of $\exp\left[j\left(\omega t-k_{z}z\right)\right]$ , where *ω* and *k*_*z*_ indicate the angular frequency and propagation constant in the *z*-direction, respectively. Each component of the electric field ***E*** and magnetic field ***H*** can be denoted by *E*_*i*_ and *H*_*i*_, respectively, where *i* = *x*, *y*, *z*. As the focus was on TM waves propagating in the *z*-direction, *E*_*x*_ = *H*_*y*_ = 0 holds. Solving the Helmholtz equation $\partial^{2}H_{x}/\partial y^{2}=-\left(\varepsilon \mu_{0}\omega^{2}-k_{z}^{2}\right)H_{x}$ with the Maxwell equation −*∂H*_*x*_/*∂y* = *ɛ∂E*_*z*_/*∂t* under boundary conditions where *E*_*z*_ = 0 at *y* = 0 and *E*_*z*_ and *H*_*x*_ are continuous at *y* = *d*, we obtained the *x*-component of the magnetic fields inside (0 ≤ *y* ≤ *d*) and outside (*y* ≥ *d*) the waveguide as follows:

(1) $$H_{x}=\left\{\begin{array}{cc} A_{\text{in}}\cos\left(k_{y}y\right)\exp\left[j\left(\omega t-k_{\text{z}}z\right)\right] & \left(0\leq y\leq d\right),\\ A_{\text{in}}\sin\left(k_{y}d\right)\exp\left[-\kappa_{y}\left(y-d\right)\right]\exp\left[j\left(\omega t-k_{z}z\right)\right] & \left(y>d\right). \end{array}\right.$$

Here, *k*_*y*_, *κ*_*y*_, and *A*_in_ denote the wave vector in the *y*-direction inside the waveguide, the extinction coefficient outside the waveguide, and the complex amplitude of the mode, respectively. The other components of the electric fields, *E*_*y*_ and *E*_*z*_, can be calculated using the Maxwell equations as −*∂H*_*x*_/*∂y* = *ɛ∂E*_*z*_/*∂t* and *∂H*_*x*_/*∂z* = *ɛ∂E*_*y*_/*∂t*, respectively. The continuity of *H*_*x*_ at *y* = *d* led to the following relationship between *k*_*y*_ and *κ*_*y*_:

(2) $$k_{y}=\varepsilon_{\text{s}}\kappa_{y}\cot\left(k_{y}d\right).$$

Equation (2) with $k_{z}^{2}+k_{y}^{2}=\varepsilon_{\text{s}}k_{0}^{2}$ and $k_{z}^{2}-\kappa_{y}^{2}=k_{0}^{2}$ yields the dispersion relation of the TM mode in the single-metalized waveguide, where *k*_0_ = *ω*/*c*_0_ and $c_{0}=1/\sqrt{\varepsilon_{0}\mu_{0}}$ .

Subsequently, we examined the scenario where the upper surface of the semiconductor waveguide is optically excited (resulting in a double-metalized waveguide, as shown in Figure 1(B)). For simplicity, the photoexcited top surface was also treated as a PEC, and its thickness was disregarded. The magnetic field in the *x*-direction for the *l*th mode $H_{x}^{\left(l\right)}$ was deduced from the Helmholtz equation under the boundary conditions *E*_*z*_ = 0 at *y* = 0 and *y* = *d* as follows:

(3) $$H_{x}^{\left(l\right)}=A^{\left(l\right)}\cos\left(k_{y}^{\left(l\right)}y\right)\exp\left[j\left(\omega^{\left(l\right)}t-k_{z}z\right)\right]\left(0\leq y\leq d\right),$$

where $A^{\left(l\right)}$ , $\omega^{\left(l\right)}$ , and $k_{y}^{\left(l\right)}=l\pi /d$ denote the complex amplitude, angular frequency, and *y*-component of the wave vector of the *l*th mode, respectively. The following dispersion relation should be satisfied:

(4) $${\left(k^{\left(l\right)}\right)}^{2}=\varepsilon_{\text{s}}\varepsilon_{0}\mu_{0}{\left(\omega^{\left(l\right)}\right)}^{2}={\left(k_{y}^{\left(l\right)}\right)}^{2}+k_{z}^{2}.$$

The condition *l* = 0 represents the TEM mode, characterized by a uniform electric field distribution in the *y*-direction ( $k_{y}^{\left(0\right)}=0$ ). Figure 1(C) depicts the dispersion curves calculated for both single- and double-metalized GaAs waveguides. The dispersion curves of *l* = 0 and *l* ≥ 1 are situated below and above that of the single-metalized waveguide, respectively. When photoexcitation transforms the structure from single-to double-metalized, the region available for electromagnetic wave propagation narrows, favoring the emergence of higher frequency modes (*l* ≥ 1). However, the TM mode in the single-metalized waveguide can engage with the TEM mode (*l* = 0) at the temporal boundary, facilitating frequency down-conversion. Conversely, frequency down-conversion is unattainable when the incident terahertz wave is polarized in the *x*-direction (TE polarization) because the TE mode cannot couple with the TEM mode [24].

### 2.2 Mode coupling and frequency conversion efficiency at the temporal boundary

The frequency conversion efficiency was evaluated by examining the mode coupling at the temporal boundary. For time *t* < 0, the electromagnetic wave was assumed to propagate in the single-metalized waveguide as the TM mode. At *t* = 0, the top surface of the semiconductor waveguide was excited by an intense laser pulse, transforming it into a double-metalized waveguide. This double-metalized state persisted for *t* > 0, and the electromagnetic waves in the waveguide conformed to the dispersion relation of the double-metalized configuration. Assuming that the refractive index inside the semiconductor (0 ≤ *y* ≤ *d*) where the terahertz waves propagate does not change across the temporal boundary, the electric and magnetic fields in the waveguide were continuous at *t* = 0. The similar assumption has been used in a theoretical analysis of the frequency conversion of TE modes in a parallel-plate waveguide whose plate spacing varies in time [28]. The electric fields in the single-metalized waveguide at *t* = −0 were distributed to each mode of the double-metalized waveguide at *t* = +0. After the temporal boundary (*t* > 0), the *x*- and *y*-components of the magnetic and electric fields propagating in the ±*z*-directions were described, considering the Fourier series of those in the double-metalized waveguide (Equation (3)), as follows:

(5) $$\begin{array}{ll} H_{x}^{\pm } & =\frac{1}{2}\left(A_{\pm }^{\left(0\right)}\right)\exp\left[j\left(\pm \omega^{\left(0\right)}t-k_{z}z\right)\right]\\ & \;+\sum_{l=1}^{\infty }A_{\pm }^{\left(l\right)}\cos\left(k_{y}^{\left(l\right)}y\right)\exp\left[j\left(\pm \omega^{\left(l\right)}t-k_{z}z\right)\right], \end{array}$$

(6) $$\begin{array}{ll} E_{y}^{\pm } & =\mp \frac{k_{z}}{\omega^{\left(0\right)}\varepsilon_{\text{s}}\varepsilon_{0}}\left(\frac{1}{2}A_{\pm }^{\left(0\right)}\right)\exp\left[j\left(\pm \omega^{\left(0\right)}t-k_{z}z\right)\right]\\ & \;\mp \overset{\infty }{\underset{l=1}{\sum }}\frac{k_{z}}{\omega^{\left(l\right)}\varepsilon_{\text{s}}\varepsilon_{0}}A_{\pm }^{\left(l\right)}\cos\left(k_{y}^{\left(l\right)}y\right)\exp\left[j\left(\pm \omega^{\left(l\right)}t-k_{z}z\right)\right], \end{array}$$

where $A_{\pm }^{\left(l\right)}$ represents the complex amplitudes of $H_{x}^{\pm }$ . Here, *k*_*z*_ was conserved before and after the temporal boundary, and the amplitude of the electromagnetic waves with *ω* was distributed to those with $\omega^{\left(l\right)}$ . The frequency was then converted from *ω* to $\omega^{\left(l\right)}$ at the temporal boundary. Based on the assumption of continuity of *E*_*y*_ and *H*_*x*_ at *t* = 0,

(7) $$\begin{array}{ll} A_{\text{in}}\cos\left(k_{y}y\right) & =\frac{1}{2}\left(A_{+}^{\left(0\right)}+A_{-}^{\left(0\right)}\right)\\ & \;+\sum_{l=1}^{\infty }\left(A_{+}^{\left(l\right)}+A_{-}^{\left(l\right)}\right)\cos\left(k_{y}^{\left(l\right)}y\right), \end{array}$$

(8) $$\begin{array}{ll} \frac{1}{\omega }A_{\text{in}}\cos\left(k_{y}y\right) & =\frac{1}{\omega^{\left(l\right)}}\frac{1}{2}\left(A_{+}^{\left(0\right)}-A_{-}^{\left(0\right)}\right)\\ & \;+\overset{\infty }{\underset{l=1}{\sum }}\frac{1}{\omega^{\left(l\right)}}\left(A_{+}^{\left(l\right)}-A_{-}^{\left(l\right)}\right)\cos\left(k_{y}^{\left(l\right)}y\right), \end{array}$$

were obtained. The relationship between the complex amplitudes of the incident wave and the *l*th order mode was reduced by multiplying both sides of Equations (7) and (8) by $\cos\left(k_{y}^{\left(l\right)}y\right)$ and integrating over *y* = 0 → *d* as

(9) $$\frac{A_{\pm }^{\left(0\right)}}{2A_{\text{in}}}=\frac{1}{2}\left(1\pm \frac{\omega^{\left(0\right)}}{\omega }\right)\text{sinc}\left(k_{y}d\right),$$

(10) $$\begin{array}{ll} \frac{A_{\pm }^{\left(l\right)}}{A_{\text{in}}} & =\frac{1}{2}\left(1\pm \frac{\omega^{\left(l\right)}}{\omega }\right)\left[\text{sinc}\left\{\left(k_{y}^{\left(l\right)}+k_{y}\right)d\right\}\right.\\ & \;\left.+\text{sinc}\left\{\left(k_{y}^{\left(l\right)}-k_{y}\right)d\right\}\right], \end{array}$$

where $\text{sinc}\left(x\right)\equiv \sin\left(x\right)/x$ . Equations (9) and (10) represent the amplitude coupling coefficients of TEM (*l* = 0) and TM (*l* ≠ 0) modes in the double-metalized waveguide, respectively.

The frequency conversion efficiency can be calculated using Equations (9) and (10). The energy of each mode can be obtained by integrating the time-averaged electromagnetic wave energy density in the *y*-direction as $w=\int_{0}^{\infty }\frac{\mu_{0}}{4}H_{x}H_{x}^{*}dy+\int_{0}^{\infty }\frac{\varepsilon }{4}\left(E_{y}E_{y}^{*}+E_{z}E_{z}^{*}\right)dy$ . We considered the energy of each mode after the temporal boundary $w^{\left(l\right)}$ and that of the TM mode of the single-metalized waveguide before the temporal boundary *w*_in_. Consequently, the frequency conversion (power transmission) coefficients $T_{l}=w^{\left(l\right)}/w_{\text{in}}$ of the TEM (*l* = 0) and TM (*l* ≠ 0) modes in the double-metalized waveguide can be deduced as

(11) $$\begin{array}{ll} T_{0} & =\frac{w^{\left(0\right)}}{w_{\text{in}}}\\ & ={\left(\frac{A_{+}^{\left(0\right)}}{2A_{\text{in}}}\right)}^{2}\frac{2}{1+\frac{k_{z}^{2}}{k^{2}}\text{sinc}\left(2k_{y}d\right)+\frac{1}{\kappa_{y}d}\frac{k_{z}^{2}}{k_{0}^{2}}\cos^{2}\left(k_{y}d\right)}, \end{array}$$

(12) $$\begin{array}{ll} T_{l} & =\frac{w^{\left(l\right)}}{w_{\text{in}}}\\ & ={\left(\frac{A_{+}^{\left(l\right)}}{A_{\text{in}}}\right)}^{2}\frac{1}{1+\frac{k_{z}^{2}}{k^{2}}\text{sinc}\left(2k_{y}d\right)+\frac{1}{\kappa_{y}d}\frac{k_{z}^{2}}{k_{0}^{2}}\cos^{2}\left(k_{y}d\right)}, \end{array}$$

where $k=\sqrt{\varepsilon_{\text{s}}}k_{0}$ . Figure 1(D) depicts the plots of *T*_0_ and *T*_1_ for the aforementioned waveguide. At frequencies below approximately 0.2 THz, the conversion efficiency was low because most of the electromagnetic fields of the lowest order mode in the single-metalized waveguide existed outside the waveguide (*y* > *d*). For the input in the TM modes with frequencies of 0.35, 0.42, and 0.48 THz used in the experiment, *T*_0_ = 0.69, 0.73, and 0.75 of energy were converted to the TEM (*l* = 0) mode and *T*_1_ = 0.23, 0.21, and 0.21 were converted to the TM (*l* = 1) mode, respectively. The residuals were converted to the higher modes (*l* ≥ 2). Therefore, the primary scattering involved frequency down-conversion for the incident TM mode. In this analysis, the change occurs solely in the boundary condition at the semiconductor waveguide surface at the temporal boundary; the refractive index of the medium through which the electromagnetic waves propagate remains unchanged. Given the absence of a refractive index alteration, the electromagnetic fields are considered continuous across the temporal boundary. This continuity suggests that the total energy is conserved across the temporal boundary, a concept further elaborated in the Supplementary Material.

## 3 Experiment

**Figure 2:**
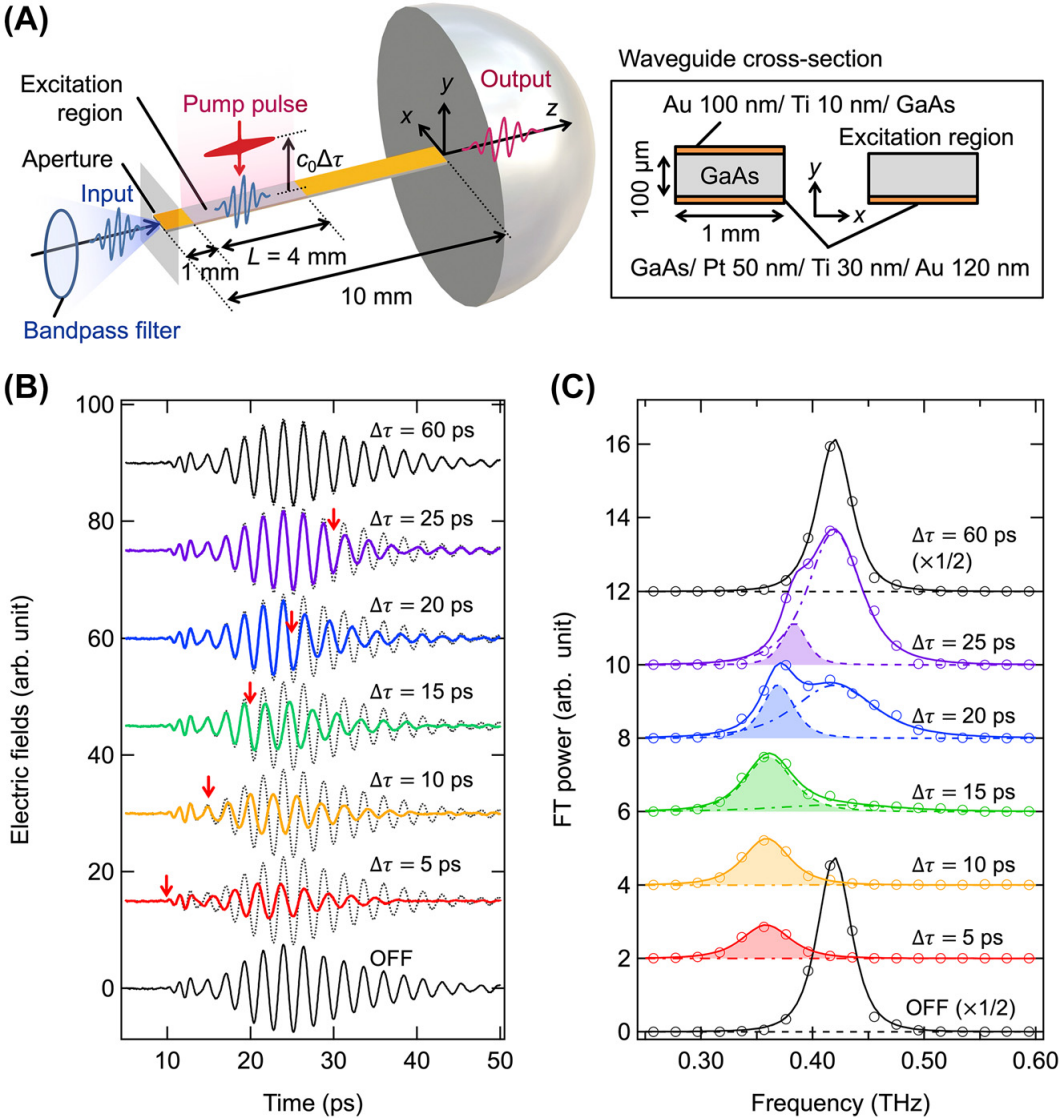
Experimental setup and observed terahertz waves. (A) Schematic of the GaAs waveguide and the experimental setup. (B) Time-domain waveforms of the terahertz wave packets passing through the waveguide with and without the pump pulses (solid lines). Each electric field waveform is offset for varying pump timing Δ*τ*. The waveforms without pump laser pulses are superimposed on each waveform with the dotted lines. The start time of the waveform changes is indicated by the red arrows. (C) Fourier-transformed power spectra (open circles) of the waveforms in panel (B). Solid lines indicate the curves fitted with the two Lorentzian functions of Equation (13). Dot-dashed and dashed lines indicate the spectral components before and after the frequency conversion in the fitted curves, respectively. The areas of the frequency-converted components are shaded.

This section outlines the experimental methodology employed for frequency down-conversion. Figure 2(A) presents a schematic representation of the waveguide and the experimental arrangement. The waveguide consisted of semi-insulating GaAs, measuring 100 μm in thickness, 10 mm in length, and 1 mm in width. The entire bottom surface of the waveguide was coated with a metal film (GaAs/Pt 50 nm/Ti 30 nm/Au 120 nm) deposited by sputtering. Additionally, a 1-mm-long metal film (GaAs/Ti 10 nm/Au 100 nm) was sputtered onto the top surface starting from the entrance edge of the waveguide. The end of the top surface was similarly covered by a metal film (GaAs/Ti 10 nm/Au 100 nm) extending to the exit edge, leaving a 4 mm long bare region. This bare area undergoes photoexcitation, altering the waveguide structure as depicted in Figure 1(A) and (B). The application of the metal film on the top surface aids in precisely defining the region subjected to optical pumping. The metal coating at the waveguide’s end serves to temporally separate the terahertz waves not engaged with the waveguide mode from those to be analyzed, thus minimizing unwanted interference.

For the realization of frequency conversion, the waveguide surface must be photoexcited while the terahertz wave traverses the optical excitation region shown in Figure 2(A). An optical-pump terahertz-probe spectroscopy system was utilized to manage the timing between the terahertz wave packet and the optical pulses. Laser pulses from a regenerative amplifier Ti:Sapphire femtosecond laser system (Spectra-Physics, Spitfire™; center wavelength = 800 nm, full-width-at-half-maximum (FWHM) = 18 nm, and pulse width ∼ 100 fs) were divided into three paths dedicated to terahertz pulse generation, detection, and sample excitation. The terahertz pulses were generated through a nonlinear optical effect in a Mg-doped LiNbO3 prism employing the tilted-pulse-front technique [29]. A double bandpass filter was used to narrow the spectral bandwidth of the terahertz pulses before reaching the waveguide sample, facilitating a clearer observation of frequency changes. As depicted in Figure 2(A), TM polarized (y-polarized) terahertz wave packets were focused at the entrance of the waveguide to initiate the TM modes.

A metal aperture, measuring 1.10 mm by 0.13 mm, was positioned near the entrance to filter out terahertz waves that did not enter the waveguide. The top surface of the waveguide was then excited by the laser pulse with a time delay Δ*τ* relative to the terahertz wave packet. In GaAs, the conduction electrons are excited by the laser pulse in less than a picosecond [30] and remain for a carrier lifetime of several hundred picoseconds [31]; consequently, the waveguide, post-optical excitation, was treated as double-metalized for terahertz waves with frequencies below the plasma frequency of the excited region. A hemispherical silicon lens was affixed to the waveguide’s end to enhance the coupling efficiency between the waveguide and air. The terahertz waves transmitted through the waveguide were then refocused onto a 3-mm-thick electro-optic crystal (ZnTe (110)) for electro-optic sampling. By varying the optical delay of the probe pulse, the time-domain electric field oscillation of the terahertz waves was observed.

## 4 Results and discussion

### 4.1 Conversion frequency

Figure 2(B) displays the time-domain waveforms of the terahertz wave packet as it passes through the waveguide at different time delays Δ*τ* of the pump pulses. Each waveform is superimposed on the waveform obtained without pump pulse irradiation (dotted lines) for comparison. The pump laser fluence was set at 0.186 mJ/cm^2^ and the center frequency of the terahertz wave packet input through the bandpass filter into the waveguide was 0.42 THz. The waveform altered by the presence of pump pulses is distinguishable from the one without them. Notably, the onset of the waveform, as highlighted by the red arrows in Figure 2(B), is delayed as Δ*τ* is adjusted. The pump timing Δ*τ* = 0 corresponds to approximately *t* = 5 ps in the time-domain waveform.

As observed for Δ*τ* larger than 10 ps in Figure 2(B), the forward portions of the terahertz wave packets that exited the optical excitation region before the pump timing exhibited no waveform alterations. The open circles in Figure 2(C) represent the Fourier-transformed power spectra. The variation in the time-domain waveform, induced by photoexcitation, manifests as a spectral peak at a frequency below the input frequency of 0.42 THz, signifying frequency down-conversion. To distinguish the frequency-converted components from the non-converted ones, the spectra in Figure 2(C) were modeled using two Lorentzian functions. Given the incident terahertz waves’ passage through a double bandpass filter, the square of the Lorentzian function was employed for the fit:

(13) $$g\left(f\right)={\left(\frac{A_{\text{a}}}{{\left(f-f_{\text{a}}\right)}^{2}+B_{\text{a}}^{2}}\right)}^{2}+{\left(\frac{A_{\text{b}}}{{\left(f-f_{\text{b}}\right)}^{2}+B_{\text{b}}^{2}}\right)}^{2},$$

where *A*_a_ (*A*_b_), *f*_a_ (*f*_b_), and *B*_a_ (*B*_b_) represent the amplitude, resonant frequency, and width, respectively. The subscripts a and b indicate the components after and before frequency conversion, respectively. The two spectral components are assumed to overlap without interference. The solid lines in Figure 2(C) represent fitting with *f*_b_ fixed to *f*_in_ = 0.42 THz, and fit well with the experimental spectra. The spectral components before and after the frequency conversion are separated into the dot-dashed and dashed lines in Figure 2(C), respectively. For Δ*τ* = 5 and 10 ps, the frequency was down-converted from the input frequency (*f*_in_) of 0.42 THz to a converted frequency (*f*_out_) of 0.36 THz. For Δ*τ* larger than 10 ps, the spectra contain components from the input frequency (0.42 THz), and two prominent peaks are observed at Δ*τ* = 20 ps. At Δ*τ* = 20 and 25 ps, the frequency-converted components are slightly shifted to higher frequencies. This may be due to interference between the frequency-converted and non-converted components. At Δ*τ* = 60 ps, no frequency conversion occurs as the pump pulse arrives after the terahertz wave packet has completely emerged from the optical excitation region. The black square in Figure 1(C) denotes the input frequency *f*_in_ = 0.42 THz plotted on the dispersion curve of the single-metalized waveguide. The converted frequency *f*_out_ = 0.36 THz is plotted as red square in Figure 1(C), assuming the wavenumber *k*_*z*_ is conserved during frequency conversion. When the bandpass filter was changed and *f*_in_ was set to 0.35 and 0.48 THz, *f*_out_ was 0.28 and 0.41 THz, respectively. These are also plotted in Figure 1(C) as the black circle and triangle (*f*_in_), and the red circle and triangle (*f*_out_). The measured converted frequencies concur with the dispersion curve of the TEM mode (*l* = 0) in the double-metalized waveguide, as was theoretically predicted. However, a frequency up-conversion to the TM mode from 0.42 THz to 0.56 THz (*l* = 1) was also predicted but was not experimentally observed (Figure 2(C)), attributed to propagation losses. This aspect will be further discussed in later sections.

### 4.2 Power ratio of the frequency-converted terahertz waves

**Figure 3:**
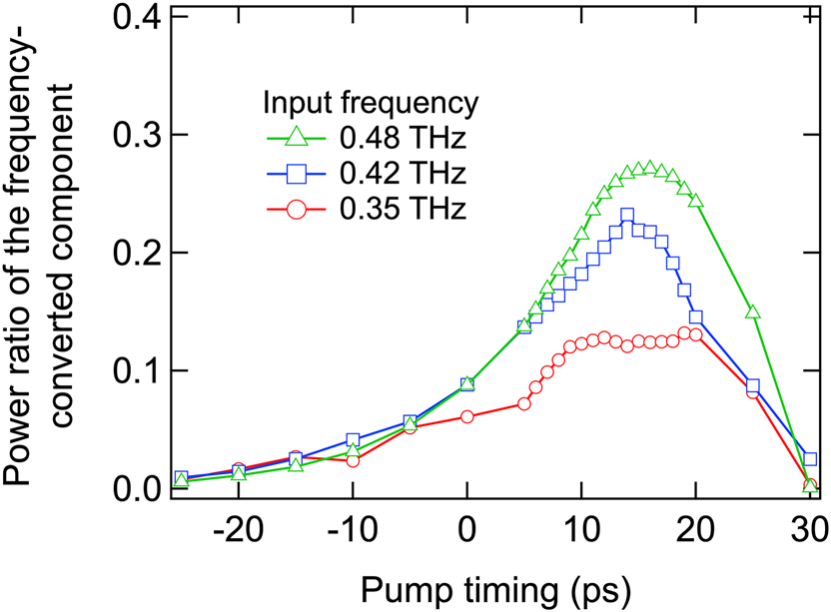
Power ratios of the frequency-converted components for input frequencies of 0.35 THz (red circles), 0.42 THz (blue squares), and 0.48 THz (green triangles).

The proportion of power from the incident terahertz wave packet that underwent frequency conversion at the temporal boundary is deduced from the fitting curves in Figure 2(C). The power ratio of the frequency-converted component relative to the terahertz waves impinging on the temporal boundary was determined by the ratio of the integrals of the frequency-converted spectral component (indicated by the shaded area in Figure 2(C)) to that in the absence of photoexcitation (OFF state in Figure 2(C)). Figure 3 presents the power ratio of the frequency conversion component as a function of the pump timing Δ*τ* for *f*_in_ = 0.35 (red circles), 0.42 (blue squares), and 0.48 THz (green triangles). The power ratio increases with Δ*τ* and reaches a maximum around Δ*τ* = 15 ps. This suggests that the optimal moment for optical excitation, coinciding with the main portion of the terahertz wave packet reaching the edge of the optical excitation region, yields the highest power ratio for the frequency-converted components. The peak power ratios for the frequency-converted components are 0.13, 0.23, and 0.27 for *f*_in_ = 0.35, 0.42, and 0.48 THz, respectively.

### 4.3 Estimation of attenuation coefficient

**Figure 4:**
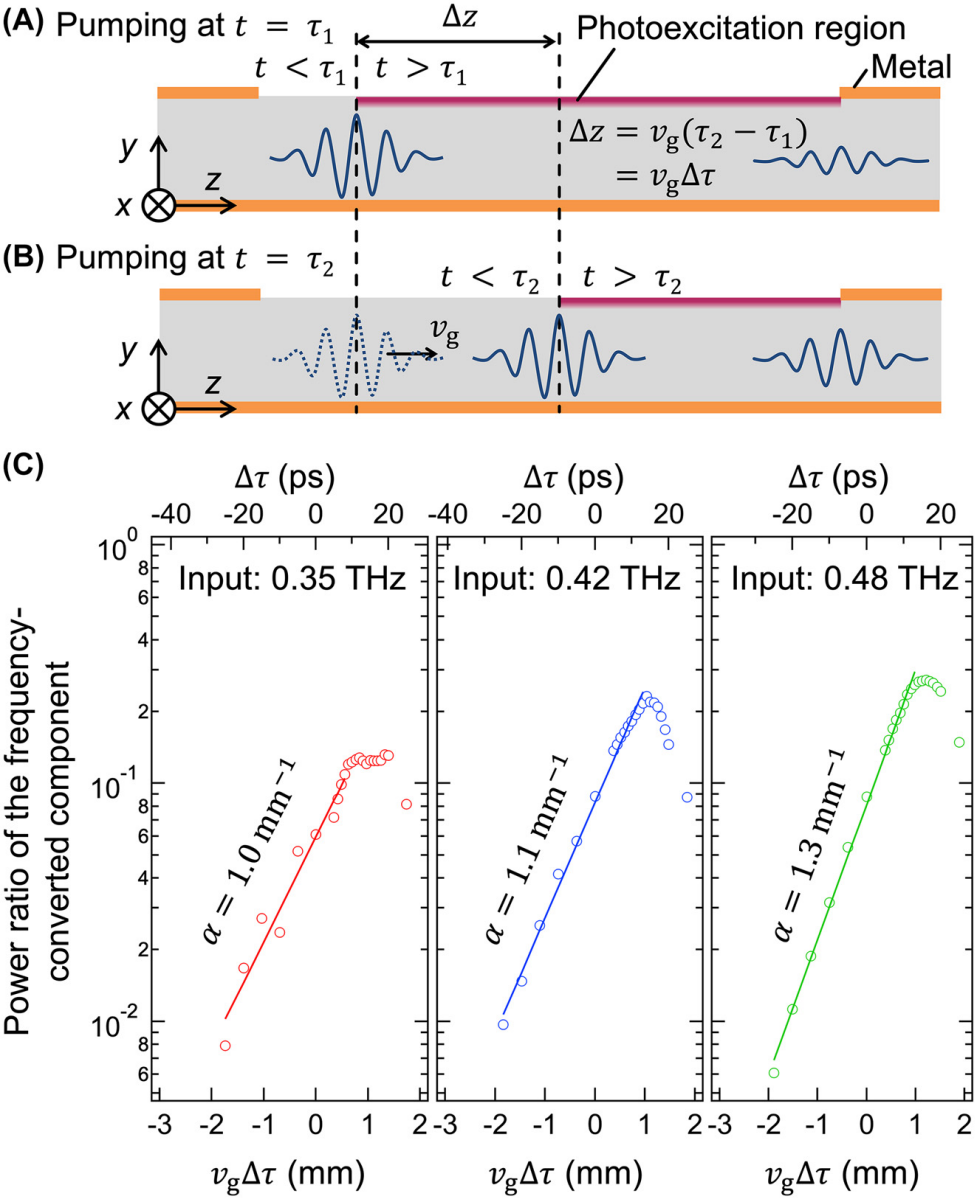
Schematics of the propagation of the terahertz wave packet when the pump pulse arrives at (A) *t* = *τ*_1_ and (B) *t* = *τ*_2_. (C) Power ratios of the frequency converted components for input frequencies of 0.35, 0.42, and 0.48 THz. The open circles and solid lines indicate the experimental results and fitting curves in the form of *A* exp[−*α*(−Δ*z*)], respectively.

After passing through the temporal boundary, the terahertz waves were presumed to experience attenuation until they emerged from the photoexcitation region. To substantiate this hypothesis, we analyzed the power attenuation coefficient *α* for the frequency-converted terahertz waves based on the following experimental observations. The pump timing Δ*τ* was correlated with the propagation distance of the terahertz wave packet in the waveguide subsequent to photoexcitation. Assuming the waveguide was excited at *t* = *τ*_1_, the frequency-converted terahertz wave packet propagated beneath the photoexcited GaAs surface from *t* = *τ*_1_ until it reached the end of the photoexcitation region (Figure 4(A)).

When the waveguide was excited at *t* = *τ*_2_ (Figure 4(B)), the propagation distance beneath the photoexcited surface was reduced by $\Delta z=v_{\text{g}}\left(\tau_{2}-\tau_{1}\right)=v_{\text{g}}\Delta \tau$ compared to that at *t* = *τ*_1_. Here, *v*_g_ denotes the group velocity in the single metalized waveguide before the temporal boundary. Consequently, the power attenuation coefficient *α* can be calculated by fitting the power ratio to the form $A\exp\left[-\alpha \left(-\Delta z\right)\right]$ . Figure 4(C) illustrates the power ratio plotted as a function of Δ*z* = *v*_g_Δ*τ*; group velocities of *v*_g_ = 7.0 × 10^7^, 7.4 × 10^7^, and 7.6 × 10^7^ m/s were applied for *f*_in_ = 0.35, 0.42, and 0.48 THz, respectively. The solid lines in Figure 4(C) depict the fitting in the region where the power ratios of the frequency-converted components demonstrate a linear increase on a logarithmic scale. The absorption coefficients were determined to be 1.0, 1.1, and 1.3 mm^−1^ for *f*_in_ = 0.35, 0.42, and 0.48 THz, respectively. As the power ratio in the experiment was normalized to the power without the pump pulse, attenuation effects not affected by the photoexcitation were omitted from the attenuation coefficient *α*. The attenuation coefficient in the single-metalized waveguide without optical pumping was considered negligible compared to the obtained *α* because the conductivity of Au is as high as 3.1 × 10^7^ S/m [32] in the terahertz frequency range and the power absorption coefficient of GaAs is lower than approximately 0.05 mm^−1^ below 1 THz [33], as estimated in the Supplementary Material.

### 4.4 Validating the theoretical prediction

**Figure 5:**
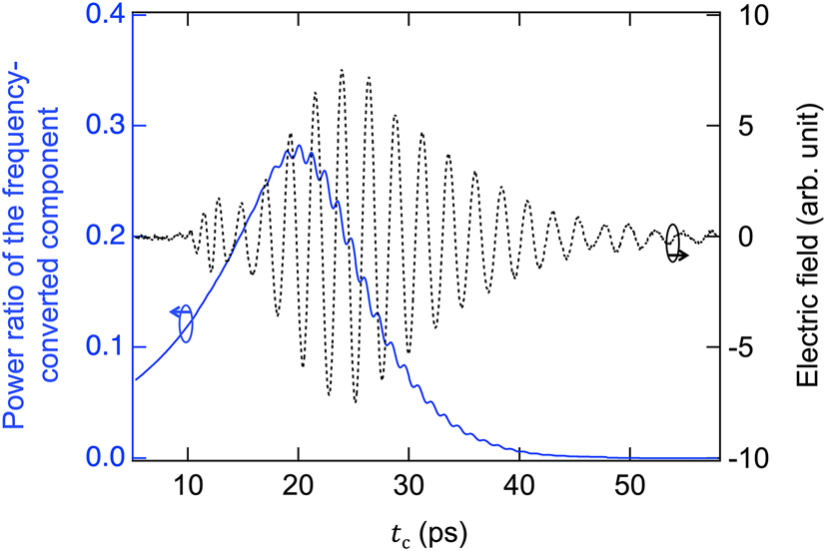
Calculation of the power ratio (solid blue line) of the frequency-converted component for *f*_in_ = 0.42 THz when photoexcited at time *t*_c_ on the time-domain waveform. The time-domain waveform for the calculation is superimposed as the dotted black line.

The power ratios of the frequency-converted terahertz waves, as estimated from Figure 3, were approximately 20 %–40 % of the predicted frequency conversion efficiency shown in Figure 1(D). This discrepancy between the power ratio and the conversion efficiency can be attributed to propagation loss, characterized by the attenuation coefficient *α*, thus corroborating the accuracy of the proposed frequency conversion efficiency model. Given that the propagation distance in the waveguide following frequency conversion is contingent upon the timing of optical excitation, the power ratio of the frequency-converted components is influenced by the pump timing, as depicted in Figure 3. If the waveguide surface is subjected to optical pumping at time *t*_c_ within the time-domain waveform of the terahertz wave packet, the waves after *t*_c_ undergo frequency conversion and subsequently experience attenuation attributed to *α* as they traverse the optical excitation region. Assuming a theoretical conversion efficiency *T*_0_ of 0.73 and an experimentally derived attenuation coefficient *α* of 1.1 mm^−1^ for *f*_in_ = 0.42 THz, the power ratio of the frequency-converted component relative to the pump time *t*_*c*_ can be calculated from the time-domain waveform absent of optical pumping, as demonstrated in Figure 5. The methodology for the calculation presented in Figure 5 is elaborated upon in the Supplementary Material. The dotted line in Figure 5 represents the time-domain waveform (without photoexcitation) overlaid with the power ratio. The peak power ratio of the frequency-converted component, achieved at *t*_c_ = 20 ps, which corresponded to Δ*τ* ∼ 15 ps. The maximum power ratio of the frequency-converted components of 0.29 in Figure 5 aligns with the value of 0.23 obtained from Figure 3. This result indicates that the observed efficiency of the frequency conversion process at the temporal boundary is consistent with the theoretical prediction, thereby validating the formulation of the frequency conversion derived from the continuity of the electric and magnetic fields at the temporal boundary.

### 4.5 Dependence of power ratio on the pump pulse fluence

**Figure 6:**
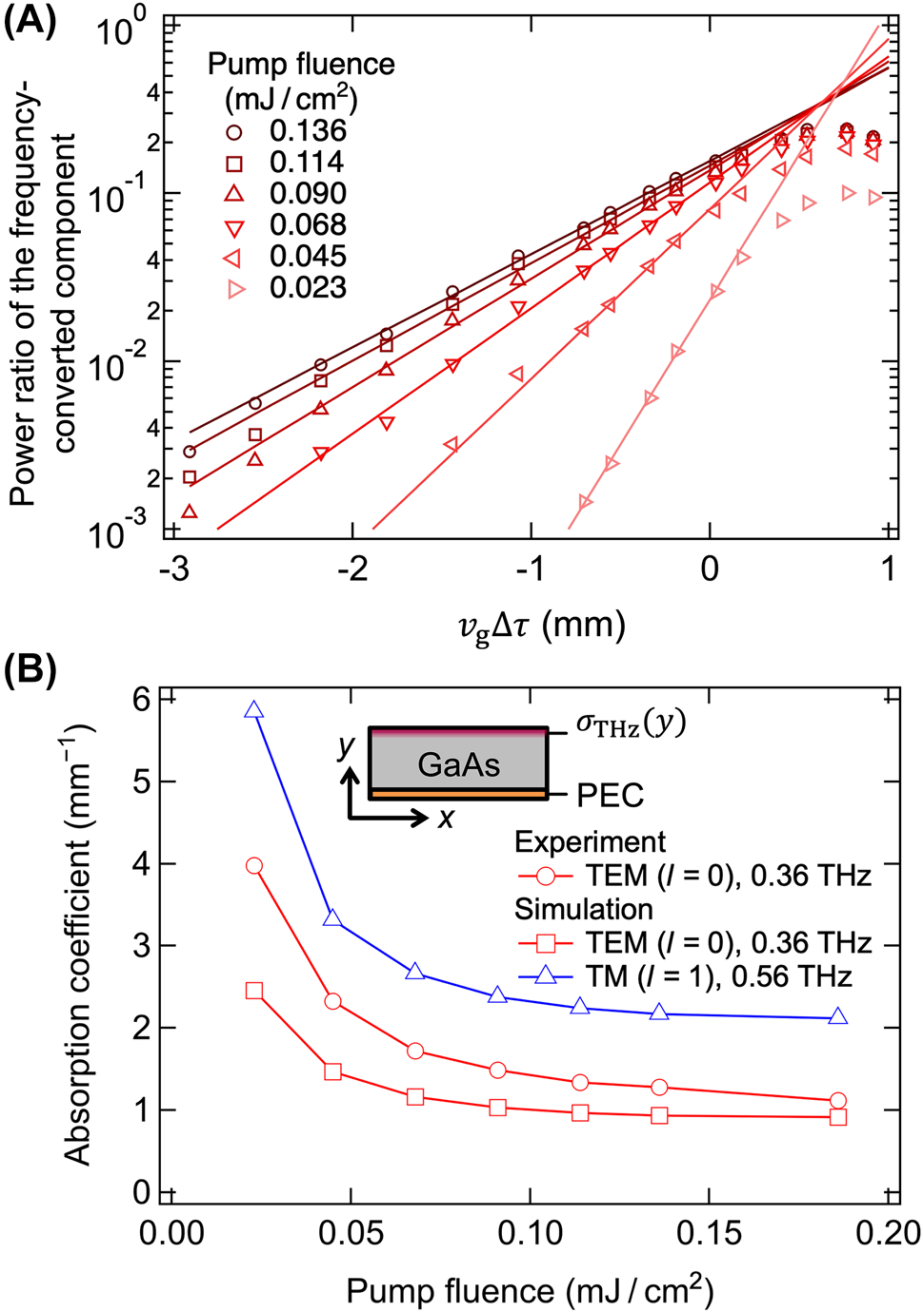
Impact of pump pulse fluence on power and attenuetion coefficients of frequency-converted terahertz waves. (A) Power ratio of the frequency-converted terahertz waves with and without the pump pulse when its fluence is varied. The lines are fitted with exponential functions. (B) Attenuation coefficients obtained from the experiment and simulation. The experimental results are obtained from the fitting lines in panel (A) and Figure 4(C). The inset represents the schematic cross-section of the simulation model. A two-dimensional waveguide whose bottom surface was defined as a perfect electric conductor (PEC) and the upper surface was defined to exhibit the conductivity distribution described in Figure S3(B) and Equation (14).


The observed increase in the attenuation coefficient with increasing frequency, as depicted in Figure 4(C), indicative of heightened Ohmic loss, led to an investigation into the photoconductivity at the waveguide surface to comprehend its influence. The absorption coefficients resulting from photoconductivity were modeled using the physical characteristics of GaAs and compared with the attenuation coefficients derived from the experiment. Moreover, this absorption explained the non-observation of frequency up-conversion in the experimental results. The conductivity can be modulated by adjusting the fluence of the pump laser pulse. Figure 6(A) illustrates the power ratio for *f*_in_ = 0.42 THz as a function of Δ*z* for varying pump pulse fluences; no frequency-converted wave was detected below a fluence of 0.023 mJ/cm^2^. Additionally, the gradient of the lines on a logarithmic scale sharpens with the reduction in pump pulse fluence. The attenuation coefficients *α* obtained by fitting exponential functions to the data from Figure 6(A), are presented in Figure 6(B) as circles, exhibiting a consistent decrease with increasing pump fluence. We calculated the electron density distribution *n*_e_ and conductivity at the GaAs surface induced by the pump pulse, as detailed in the Supplementary Material. Considering the intense laser pulses’ excitation of the waveguide, the band-filling effect, which limits the number of excited electrons [34], was taken into account. With the increase in pump laser intensity, the density of excited electrons near the GaAs surface reached a saturation point at a maximum value of *n*_max_ = 1.4 × 10^18^ cm^3^ constrained by the band-filling effect (Figure S3(B) in the Supplementary Material). The contribution of two-photon absorption to the electron density was deemed negligible within the analyzed pump fluence range. Based on the electron density, the distribution of the plasma frequency *ω*_p_ and complex electrical conductivity *σ*_THz_ can be calculated using the Drude model as:

(14) $$\omega_{\text{p}}=\sqrt{\frac{n_{\text{e}}e^{2}}{m_{\text{c}}\varepsilon_{0}\varepsilon_{\text{s}}}},\;\;\;\;\;\sigma_{\text{THz}}=\frac{\varepsilon_{0}\varepsilon_{\text{s}}\omega_{\text{p}}^{2}}{\gamma +j\omega },$$

where *γ* = *e*/*m*_0_ *μ*_mov_. *m*_c_ = 0.063*m*_0_ and *μ*_mov_ = 7.8 × 10^3^ cm^2^/Vs represent the effective mass and mobility of the conduction electrons, respectively [35]. *e* and *m*_0_ denote the elementary charge and the electron mass, respectively.

The absorption coefficients of the propagation modes in a two-dimensional waveguide were determined by applying the estimated *σ*_THz_ in a finite element electromagnetic simulation (COMSOL Multiphysics^®^ 5.4). A schematic of the cross-section of the simulation model is shown in the inset of Figure 6(B). In line with the experimental approach, where the loss components unaffected by optical excitation were excluded from the attenuation coefficient *α*, the bottom of the waveguide was modeled as a PEC in the simulation. The conductivity distribution *σ*_THz_ was applied to the upper surface. Power transmittances for various waveguide lengths and propagation modes were simulated, and the power absorption coefficients for each mode were obtained through exponential fitting. The absorption coefficients for the TEM (*l* = 0 at 0.36 THz) and TM (*l* = 1 at 0.56 THz) modes were calculated, as shown in Figure 6(B). The trend of the absorption coefficient for the TEM mode, characterized by a rapid increase at low fluences and a decrease at higher fluences, aligned with the experimental observations. For example, the values of *α* were 1.1 and 0.92 mm^−1^ in the experiment and simulation, respectively, for a fluence of 0.186 mJ/cm^2^. Despite the simplified approach to estimating the absorption coefficients induced by photoconductivity, the experimentally derived values of *α* were of the same order of magnitude as those from the simulation, suggesting that conduction loss in the photoexcited region of the GaAs surface predominantly contributed to the attenuation of the frequency-converted terahertz waves. Although the scattering coefficient *γ* was presumed constant for the simulation, *γ* is known to increase with carrier density [36], which in turn decreases *σ*_THz_. The decrease in *n*_max_ caused by the defects in the GaAs crystal may also increase *α* in the experiment. Moreover, the absorption coefficient of the TM mode (*l* = 1) was nearly twice that of the TEM mode (*l* = 0), which is coupled with a low conversion efficiency (*T*_1_ = 0.21), accounts for the non-observation of frequency up-conversion to the TM mode at 0.56 THz in the experiment.

## 5 Conclusions

In this study, we have demonstrated the frequency down-conversion of TM mode terahertz waves by modifying the structure of the GaAs waveguide through optical pulse excitation. Unlike the frequency up-conversion observed with TE modes [24], TM modes can couple with the TEM mode to facilitate frequency down-conversion. The frequency conversion process was analyzed from the continuity of the electric and magnetic fields, based on the assumption that only the boundary condition of the waveguide surface varies in time. Our analysis of the experimental results confirmed that the efficiency of the frequency conversion at the temporal boundary aligns with predictions made by subtracting the propagation loss subsequent to the frequency conversion. The dominant factor in the attenuation of the frequency-converted terahertz wave was the conduction loss induced by the finite electrical conductivity of the photoexcited region. The increase in conductivity of the photoexcited region, induced by higher intensity pump pulses, is constrained by the saturation of linear absorption in GaAs. Consequently, employing semiconductor materials with narrower band gaps than GaAs and utilizing higher energy pump pulses could enhance the number of excited electrons and reduce Ohmic loss in the frequency-converted waves. Moreover, if the propagation loss is mitigated, frequency up-conversion, as indicated by Equation (12), could be simultaneously observed with frequency down-conversion for TM polarization. This would allow information to be simultaneously transferred to both the upper and lower frequency channels during frequency division multiplexing.

The efficiency of frequency conversion at the temporal boundary does not depend on the source intensity. The advantage of temporal waveguide systems lies in the capability to design the converted frequency based on the modes used and structural parameters, such as thickness. Given the frequency up-conversion for TE waves, the selection between frequency up- and down-conversion can be achieved by manipulating the input polarization for the waveguides. This suggests that the waveguide is capable of realizing temporal frequency birefringence [37]. After the temporal boundary, the effective refractive index becomes anisotropic, and the conversion frequency varies depending on the incident polarization. Achieving such birefringence at the temporal boundary is challenging in homogeneous natural materials. Techniques for manipulating the polarization of terahertz waves have been developed using metamaterials that can dynamically alter phase responses [38], [39]. Integrating these techniques with frequency conversion at the temporal birefringence boundary in the waveguide could lead to the development of essential active devices, significantly contributing to high data transfer rates in future integrated terahertz photonic circuits [23], [40].

Finally, it is important to recognize that the concept of this study extends beyond the terahertz frequency range and can be applied to the optical frequency range as well. The ultrafast modulation of the plasma frequency and the corresponding near-infrared refractive index changes in indium tin oxide through photoexcitation have been demonstrated [41], [42]. This indicates the potential for creating ultrafast frequency conversion devices comprising optically modulated waveguides with indium tin oxide.

## Supplementary Material

Supplementary Material Details
