# Supplementary material for: Frequency down-conversion of terahertz waves at optically induced temporal boundaries in GaAs waveguides
Source: Nanophotonics. 2024 May 20;13(17):3077–89. doi: 10.1515/nanoph-2024-0010 (PMC11501523; doi:10.1515/nanoph-2024-0010)
Supplement: Supplementary file 1 — Supplementary Material Details [file j_nanoph-2024-0010_suppl_001.pdf]

Supplementary Material

Frequency Down-Conversion of Terahertz Waves at Optically Induced Temporal Boundaries in GaAs Waveguides

Keisuke Takano[1], Satoko Uchiyama[1], Shintaro Nagase[1], Yuka Tsuchimoto[1], Toshihiro Nakanishi[2], Yosuke Nakata[3,4], Joel Pérez-Urquizo[5,6], Julien Madéo[5], Keshav M. Dani[5], and Fumiaki Miyamaru[1]*

[1]*Department of Physics, Faculty of Science, Shinshu University, Nagano 390-8621, Japan*

[2]*Department of Electronic Science and Engineering, Kyoto University, Kyoto 615-8510, Japan*

[3]*Graduate School of Engineering Science, Osaka University, Osaka 560-8531, Japan*

[4]*Center for Quantum Information and Quantum Biology, Osaka University, Osaka 560-0043, Japan*

[5]*Femtosecond Spectroscopy Unit, Okinawa Institute of Science and Technology Graduate University, Okinawa 904-0495, Japan*

[6]*Laboratoire de Physique de l'École Normale Supérieire ENS, Université PSL, CNRS, Sorbonne Université, Université de Paris, F-75005 Paris, France.*

*ksk_takano@shinshu-u.ac.jp

1. Power Absorption Coefficient in Single-Metalized GaAs Waveguides

The power absorption coefficient in the single-metalized GaAs waveguide was estimated using the finite element method simulation software (COMSOL Multiphysics® 5.4). **Figure S1**(A) illustrates the schematic of the simulation model. The waveguide is uniform in the x-direction, and electromagnetic propagation is considered in the z-direction. The entrance and exit of the waveguide comprise a 0.5-mm-long parallel plate. The top and bottom surfaces of the gold-composed parallel-plate waveguides have thicknesses of 100 nm and 120 nm, respectively. The electrical conductivity of gold was assumed to be 3.1×10^{7} S/m.[1] The 100-μm-thick GaAs was assumed to have a relative permittivity of 12.96 in the real part and a power absorption coefficient of 0.05 mm^{-1}.[2] A single metalized waveguide of length L was placed between the parallel-plate waveguides, as depicted in Figure S1. A TEM mode of 0.42 THz was excited at the entrance of the waveguide, and the power transmission of the electromagnetic waves propagating in the z-direction was calculated for different values of L. Figure S1(B) illustrates the power transmittance (open circles) calculated by varying the value of L and the fitting curve (solid line) in the form of $A\,\exp(-\beta L)$. The fitting indicated that the estimated power absorption coefficient β of the single-metalized waveguide was 0.054 mm^{-1}.

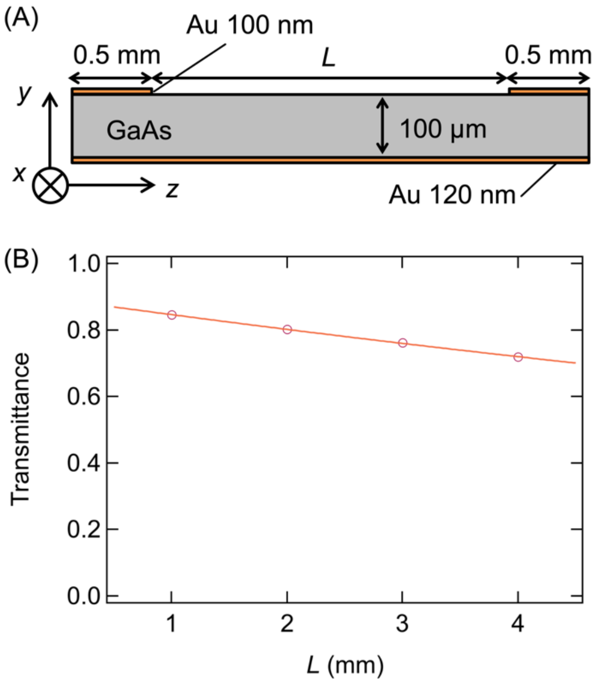

Figure S1. (A) Schematic of the simulation model to estimate the power absorption coefficient of the single-metalized GaAs waveguide. (B) Transmittance of the waveguide for different lengths L at 0.42 THz (open circles). The solid line indicates the exponential fitting curve.

2. Power Ratio of the Frequency-Converted Waves Attenuated by α

The frequency-converted terahertz waves in the waveguide were attenuated by the attenuation coefficient α until they exited the optical excitation region. We estimated the power ratio of the frequency-converted component by employing the theoretical frequency conversion efficiency T_0 and the experimental power attenuation constant α. Depending on the timing of the photoexcitation, the propagation distance of the frequency-converted components through the photoexcited region varied. To consider the propagation distance in the waveguide, the time-domain waveform without the excitation pulse (**Figure S2**(A)) was transformed into a space-domain waveform in the waveguide by multiplying the time by the phase velocity $v_{\mathrm{p}}^{\mathrm{b}}$ in the single-metalized waveguide. Here, $v_{\mathrm{p}}^{\mathrm{b}} = 1.2 \times 10^8$, 9.5×10^7, and 9.2×10^7 m/s for f_{in} = 0.35, 0.42, and 0.48 THz, respectively. The dotted line in Figure S2(B) represents the space-domain waveform for f_{in} = 0.42 THz, depicted as an example. A relative position z_{c} is defined as the boundary position between frequency conversion and non-conversion regions, indicating the exit position of the optical excitation region during the pump excitation at $t_{\mathrm{c}} = z_{\mathrm{c}}/\, v_{\mathrm{p}}^{\mathrm{b}}$ on the time-domain waveform. The assumption that multiplying the spatial waveform in non-conversion ($z < z_{\mathrm{c}}$) and conversion regions ($z \geq z_{\mathrm{c}}$) by 0 and $\sqrt{T_0}e^{-\alpha(z-z_{\mathrm{c}})/2}$, respectively, yields the space-domain waveform of the frequency-converted terahertz wave packet can be considered reasonable. In the case of z_{c} = 1.9 mm, $\alpha = 1.1\ \mathrm{mm}^{-1}$, and $T_0 = 0.73$ for f_{in} = 0.42 THz (Figure 1(D)), the function of $\sqrt{T_0}e^{-\alpha(z-z_{\mathrm{c}})/2}$ and the waveform multiplied are presented as the red and blue lines in Figure S2(b), respectively. The space-domain waveform is transformed back to the frequency-converted time-domain waveform by dividing position z by the phase velocity $v_{\mathrm{p}}^{\mathrm{a}}$ in the double-metalized waveguide. As the dispersion curve of the TEM mode is linear, the value of phase velocity was common ($v_{\mathrm{p}}^{\mathrm{a}} = 8.1 \times 10^7\ \mathrm{m/s}$) for f_{out} = 0.28, 0.36, and 0.41 THz. In the same way as we obtained Figure 3(b), the power ratios of the frequency converted waves were calculated from T_0 and α by Fourier transforming the frequency-converted time-domain waveforms, fitting with the square of the Lorentzian function, integrating the fitted curve, and normalizing with the integral of the fitted spectrum without photoexcitation. Using the theoretical frequency conversion efficiencies T_0 = 0.69, 0.73, and 0.75 and the experimentally obtained attenuation coefficients α = 1.0, 1.1, and 1.3 mm^{-1} for f_{in} = 0.35, 0.42, and 0.48 THz, respectively, the power ratios of the frequency-converted components were determined as a function of t_{c} (Figure S2(C)). The peak values for f_{in} = 0.36,

0.42, and 0.48 THz were 0.23, 0.29, and 0.29, respectively, which were comparable to the maximum power ratios of the frequency-converted components estimated as 0.13, 0.23, and 0.27, respectively (Section 4.2).

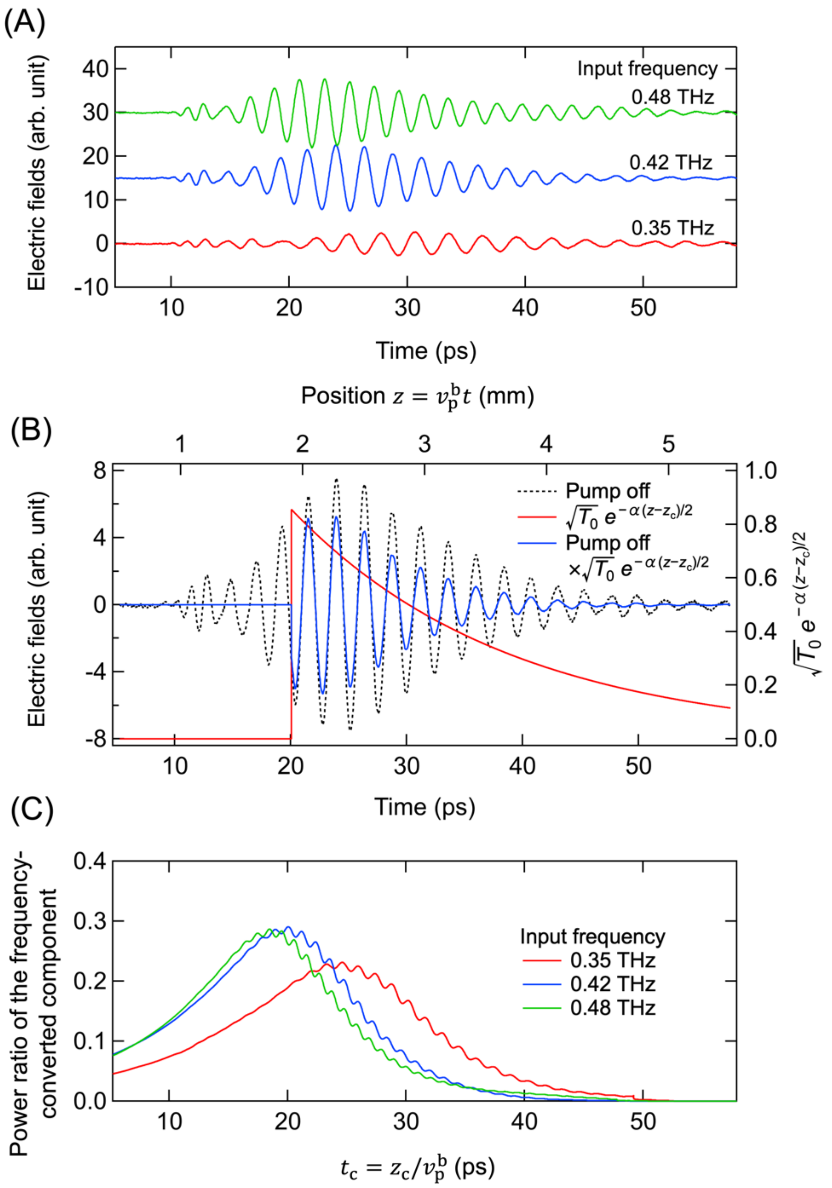

Figure S2. Estimation of the power ratios of the frequency-converted terahertz waves and the space-domain waveforms of the electric fields in the waveguide. (A) Time-domain waveforms for f_in = 0.36, 0.42, and 0.48 THz without photoexcitation. (B) The blue solid line indicates the waveform obtained by multiplying the electric field waveform without pump pulse (black dotted line) by $f(z) = \begin{cases} 0, & z < z_\mathrm{c} \\ \sqrt{T_0} e^{-\alpha(z-z_\mathrm{c})/2}, & z \geq z_\mathrm{c} \end{cases}$ (red solid line), with f_in = 0.42 THz, α = 1.1 mm^{-1}, T_0 = 0.73, and z_c = 1.9 mm. (C) Power ratio of the frequency-converted components calculated for f_in = 0.35, 0.42, and 0.48 THz.

3. Estimation of the Spatial Distribution of the Photoexcited Carrier Density

We estimated the depth profile of the photoexcited carrier density on the GaAs surface excited by intense laser pulses. In the case of intense laser pulse irradiation, the saturation of the photocarrier density occurs because of the band-filling effects. The energy width of the pump laser pulse determines the energy range to which the electron can be excited within the energy band in GaAs. The electron density that can be excited is obtained by integrating the electron density of states within that energy range. Kadlec *et al.*[3] estimated the maximum electron density resulting from the pulse laser excitation and the distribution of the excited electron density corresponding to the penetration of the laser into GaAs. Considering this, we estimated the maximum electron density n_{max} and electron density distribution n_{e} that could be excited in our experiment. **Figure S3**(A) depicts the energy band diagram of GaAs near the Γ point. During this analysis, we assumed that GaAs exhibited an energy bandgap of E_{g} = 1.42 eV,[4] and the conduction band E_{c} and valence bands E_{HH} (heavy-hole) and E_{LH} (light-hole) have parabolic band shapes. The effective masses of the conduction, heavy-, and light-hole valence bands were assumed to be isotropic, with $m_{\mathrm{c}} = 0.063m_0$, $m_{\mathrm{H}} = 0.50m_0$, and $m_{\mathrm{L}} = 0.076m_0$, respectively.[4] m_0 is the electron mass. The electrons in the valence band that existed in the energy range where the energy difference between the valence bands and conduction band ($E_{\mathrm{c}} - E_{\mathrm{HH}}$ and $E_{\mathrm{c}} - E_{\mathrm{LH}}$) matched the energy range in the full-width-at-half-maximum (FWHM) of the laser pulse (1.55 eV $\pm$ 17 meV) were excited and transited to the conduction band. The minimum and maximum of the corresponding energies in the conduction bands were $E_{\mathrm{min}} = 62$ meV and $E_{\mathrm{max}} = 131$ meV, respectively, measured from the bottom of conduction bands. When no energy redistribution of excited electrons occurred in the conduction band, the electron density that could be excited was obtained by integrating the density of conduction electron states from E_{min} to E_{max} as 2.3×10^{18} cm^{-3}. When the redistribution occurred, the electron density of states from the bottom of the conduction band to E_{max} was integrated as 3.4×10^{18} cm^{-3}. Assuming that the two aforementioned cases occurred simultaneously, the average of both integrals was used for this estimation. As no inverted distribution occurred, we estimated the maximum number of electrons that could be excited by considering half of the average value, which yielded $n_{\mathrm{max}} = 1.4 \times 10^{18}$ cm^{-3}. The optical absorption was saturated where the electron density n_{e} was close to n_{max} and the absorption coefficient was assumed to be[3]

$$a(n_e) = a_0 \frac{n_{max} - n_e}{n_{max}}, \quad \text{(S1)}$$

where $a_0 = 1.35\ \mu m^{-1}$ denotes the linear absorption coefficient of GaAs for 800 nm light.[5] The electron density distribution n_e from the surface of GaAs to a certain depth was calculated by solving the differential equation for the laser fluence [3], considering various pump fluences as depicted in Figure S3(B). Here, all absorbed photons were assumed to excite conduction electrons. As the pump laser intensity increased and the absorption coefficient decreased, n_e was saturated to n_{max} near the surface, and the laser pulse penetrated deeper into GaAs beyond the linear penetration depth $d_0 = 1/a_0$. The electron density resulting from the two-photon absorption was negligible in the range of pump fluence considered in this study. Based on the electron density, the distribution of the plasma frequency ω_p and complex electric conductivity σ_{THz} were calculated using the Drude model, as discussed in Section 4.5.

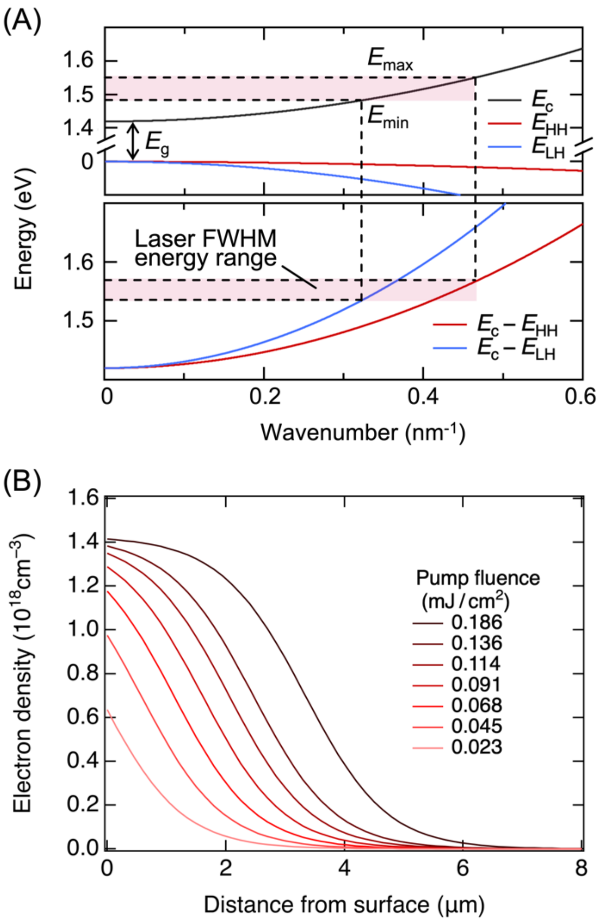

Figure S3. (A) Energy band diagram of GaAs near the Γ point. The conduction band (E_c), heavy-hole band (E_{HH}), light-hole band (E_{LH}), and their differences ($E_c - E_{HH}$ and $E_c - E_{LH}$) are presented. (B) Electron density distribution from the GaAs surface to the depth at different pump fluences.

4. Energy conservation at the temporal boundary

Typically, electromagnetic energy is not conserved in a time-varying medium when the refractive index of the medium changes and frequency shifts occur [6], [7]. This is because the energy density of the electromagnetic wave changes across the temporal changes of the refractive index. In contrast, in this study, only the boundary condition of the waveguide surface is changed by photoexcitation, and the refractive index of the medium where the electromagnetic waves propagate is not changed. If there is no refractive index change, the energy density $(\boldsymbol{E}\cdot\boldsymbol{D}+\boldsymbol{H}\cdot\boldsymbol{B})/2$ within the GaAs waveguide does not change due to the continuity of electric and magnetic fields at the temporal boundary. Thereby, the total energy is conserved as shown in Figure S4(A). Figure S4(A) illustrates the energy conversion efficiencies from the fundamental mode in the single-metalized waveguide to the l-th mode (l = 0, 1, 2) in the double-metalized waveguide at the temporal boundary. As well as Eqs. (11) and (12) in the main text, T_l and R_l represent the energy frequency conversion efficiencies to the l-th modes in the transmission and reflection directions, respectively. Figure S4(B) aggregates these efficiencies for modes l = 0 to 100, $\sum_{l=0}^{100} T_l$, $\sum_{l=0}^{100} R_l$, and $\sum_{l=0}^{100}(T_l+R_l)$. The sum of the conversion efficiencies is less than 1, particularly notable up to about 0.2 THz. This deficiency is attributed to the electromagnetic fields of the lowest-order mode of the single-metalized waveguide extending outside the GaAs waveguide, hence not coupling with the modes of the double-metalized waveguide after the temporal boundary. As the electromagnetic field components outside the GaAs waveguide diminish at higher frequencies, the sum of conversion efficiencies gradually approaches 1.

A more straightforward relationship can be obtained focusing on the electromagnetic energy within the GaAs waveguide only. By confining the integration to the interior of the GaAs waveguide and computing the time-averaged electromagnetic field energy within this semiconductor GaAs as $w^{(\mathrm{s})}=\int_0^d \frac{\mu_0}{4} H_x H_x^* dy+\int_0^d \frac{\varepsilon}{4}(E_y E_y^*+E_z E_z^*) dy$, the frequency conversion efficiency within the GaAs waveguide can be obtained. Here, d = 100 μm is the thickness of the GaAs waveguide, as illustrated in Figure 1(A). The efficiencies of frequency conversion within the GaAs waveguide, for both transmission $T_l^{(\mathrm{s})}$ and reflection $R_l^{(\mathrm{s})}$ directions, are depicted in Figure S4(C). In addition, as in Figure S4(B), the sum of them within l = 0 to 100, $\sum_{l=0}^{100} T_l^{(\mathrm{s})}$, $\sum_{l=0}^{100} R_l^{(\mathrm{s})}$, and $\sum_{l=0}^{100}(T_l^{(\mathrm{s})}+R_l^{(\mathrm{s})})$ are shown in Figure S4(D). The aggregation of these frequency conversion efficiencies within the GaAs waveguide reaches unity (indicated by the green solid line), indicating that total energy is conserved in the

frequency conversion at the temporal boundary within the waveguide. This means that the incident electromagnetic waves are redistributed to each mode after the temporal boundary while preserving the total energy.

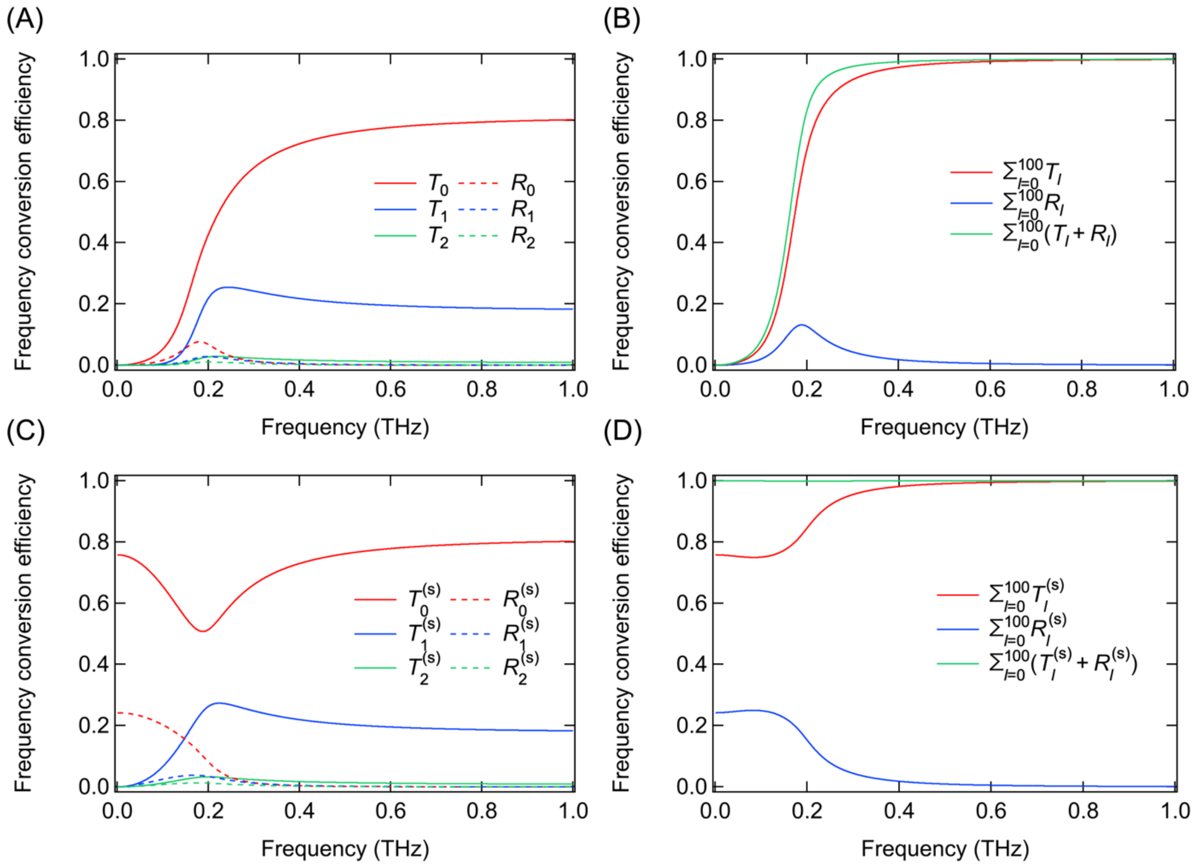

Figure S4. (A) Frequency conversion efficiency in energy from the lowest-order mode of the single-metalized waveguide to the l-th ($l = 0, 1, 2$) mode of the double-metalized waveguide at the temporal boundary. T_l and R_l represent the conversion efficiency of the components propagating in the transmission and reflection directions, respectively. (B) Sum of frequency conversion efficiencies within $l = 0$ to 100 of $\sum_{l=0}^{100} T_l$, $\sum_{l=0}^{100} R_l$, and $\sum_{l=0}^{100}(T_l + R_l)$. (C) Frequency conversion efficiency to the l-th ($l = 0, 1, 2$) modes in the transmission $T_l^{(\mathrm{s})}$ and reflection $R_l^{(\mathrm{s})}$ directions inside a waveguide when the integration range of electromagnetic energy is limited to the inside of the waveguide ($0 \le y \le d$). (D) Sum of frequency conversion efficiencies within $l = 0$ to 100, $\sum_{l=0}^{100} T_l^{(\mathrm{s})}$, $\sum_{l=0}^{100} R_l^{(\mathrm{s})}$, and $\sum_{l=0}^{100}(T_l^{(\mathrm{s})} + R_l^{(\mathrm{s})})$.

References

[1]N. Laman and D. Grischkowsky, “Terahertz conductivity of thin metal films,” Appl. Phys. Lett., vol. 93, no. 5, p. 051105, 2008, doi: 10.1063/1.2968308.

[2]D. Grischkowsky, S. Keiding, M. van Exter, and C. Fattinger, “Far-infrared time-domain spectroscopy with terahertz beams of dielectrics and semiconductors,” JOSA B, vol. 7, no. 10, pp. 2006–2015, 1990, doi: 10.1364/JOSAB.7.002006.

[3]F. Kadlec, H. Němec, and P. Kužel, “Optical two-photon absorption in GaAs measured by optical-pump terahertz-probe spectroscopy,” Phys. Rev. B, vol. 70, no. 12, p. 125205, 2004, doi: 10.1103/PhysRevB.70.125205.

[4]J. S. Blakemore, “Semiconducting and other major properties of gallium arsenide,” J. Appl. Phys., vol. 53, no. 10, pp. R123–R181, 1982, doi: 10.1063/1.331665.

[5]E. D. Palik, Handbook of Optical Constants of Solids. Elsevier, 1985. doi: 10.1016/C2009-0-20920-2.

[6]Y. Xiao, G. P. Agrawal, and D. N. Maywar, “Spectral and temporal changes of optical pulses propagating through time-varying linear media,” Opt. Lett., vol. 36, no. 4, p. 505, 2011, doi: 10.1364/OL.36.000505.

[7]M. Notomi and S. Mitsugi, “Wavelength conversion via dynamic refractive index tuning of a cavity,” Phys. Rev. A, vol. 73, no. 5, p. 051803, 2006, doi: 10.1103/PhysRevA.73.051803.
